# The 2003 Iraq War and Avoidable Death Toll

**DOI:** 10.1371/journal.pmed.1001532

**Published:** 2013-10-15

**Authors:** Salman Rawaf

**Affiliations:** WHO Collaborating Centre, Imperial College London, London, United Kingdom

## Abstract

Salman Rawaf discusses the implications of the most recent estimate of excess deaths associated with the Iraq war and subsequent occupation in the context of the current situation in Iraq.

*Please see later in the article for the Editors' Summary*

**Linked Research Article**

This Perspective discusses the following new study published in *PLOS Medicine*:

Hagopian A, Flaxman A, Takaro TK, Al Shatari SAE, Rajaratnam J, Becker S, et al. (2012) Mortality in Iraq Associated with the 2003–2011 War and Occupation: Findings from a National Cluster Sample Survey. PLoS Med 10(10): e1001533. doi:10.1371/journal.pmed.1001533

Based on a survey of 2,000 randomly selected households throughout Iraq, Amy Hagopian and colleagues estimate that close to half a million excess deaths are attributable to the recent Iraq war and occupation.

During and after disasters, the human deaths become numbers in columns for epidemiologists to discover and statisticians to analyse. In peacetime, accurate and timely mortality data serve as an important tool in population forecasting, as indicators of a population's welfare, and for planning and developing health services and programmes and evaluating them [1]–[3]. In humanitarian crises (natural and man-made), such information serves a different purpose, mainly to evaluate the severity of the crisis, both at its onset and as it evolves over time [4]. The research article by Amy Hagopian and colleagues [5] in this week's issue of *PLOS Medicine*, which provides the most recent excess mortality estimates for the 2003 Iraq invasion and subsequent occupation, is an example of the latter.

## Mortality Estimates during Conflict

Estimating mortality during conflict is difficult, and the challenges considerable. Rather than being the routine exercise that peacetime data gathering is, collection of mortality information in conflict situations requires extra efforts, using a range of methods. The gold standard, mortality surveillance (prospective death reporting), captures real-time data. But with few notable exceptions, national registration systems are barely functional during wartime [6]. Instead, surveys, in the form of retrospective data collection, are often used, although this method has known shortcomings when collecting adult mortality data [7]–[9]. A third option is demographic techniques that compare the age distribution of a population from a census taken both before and after war [10]. However, reliable census data are rarely available and often are disputed politically—and Iraq is no exception [11]. Finally, passive collection of death reports (media, eyewitness accounts, records from health facilities, and national government and international reports) is a method developed in recent years, but is widely criticised by researchers as the least reliable method of ascertaining mortality during conflict [12]–[14].

Estimating mortality during conflict is not simply a researcher's technical challenge. It is essential to quantify the catastrophe that war creates, in terms of human lives as well as damage to the environment and infrastructure. Documenting the human effects of war is a critical role of public health academics and practitioners. To the extent that such documentation can support public health advocacy and other means to avert war, and foster research to minimise war's impact on health and the environment and ameliorate its consequences, such research is much more important than a simple count of the human dead.

## Counting the Dead in Iraq

In 2003, the war in Iraq attracted worldwide interest as the systematic failure of coalition and Iraqi politicians to stabilise the country [15],[16] led to atrocities by the military and sectarian insurgents against innocent civilians. During this conflict, mortality surveys were conducted to assess the impact of the invasion and subsequent terrors. However, all have been criticised in one way or another, and almost all were perceived as being politically motivated, deliberately either over-reporting or suppressing the number of deaths [7],[17]–[19].

To many, the war in Iraq has ended, but hostilities and resulting terror continue. As recently as in July 2013, 1,057 people lost their lives, with many more injured, as a result of fresh sectarian conflict [20]. To my knowledge, none of the governments involved in the invasion or occupation have conducted an official inquiry into the health consequences of the conflict, calling into question their interest in the true impact of their actions on the civilians in Iraq. But public health advocates and researchers want to know, and the international community has a right to know. In this context, the retrospective survey by Amy Hagopian et al. is valuable. The authors attempt to overcome methodological difficulties encountered in a previous survey by Burnham and colleagues [7], criticised for its small sample size and cluster methodology, and the 2006/2007 Iraq Family Health Survey, compromised by missing data, as many of the intended sampling sites could not be reached for security reasons [19].

## The Latest Estimates: Implications for Iraq and Beyond

Given the continued lack of security, huge internal and external population displacement, as well as general lack of trust in post-invasion Iraq, it remains very difficult to conduct a reliable household study. For their survey, conducted in early 2011, Amy Hagopian and colleagues randomly selected 2,000 households in 100 clusters distributed throughout Iraq's 18 governorates. They were able to collect data from all but one cluster, which was skipped for security reasons. They inquired about deaths in two ways: they asked the head of each household about deaths among household members, and they asked all adults in the household about deaths among their siblings. Unlike earlier surveys, the researchers also added a correction for deaths that would have been reported by households that had emigrated. Covering almost the entire duration of the Iraq occupation, including, for the first time, a critical period of violence from 2006 to 2008, they conclude that approximately half a million avoidable deaths in Iraq can be attributed directly and indirectly to the invasion and subsequent occupation and related insurgencies.

This estimate carries substantial uncertainty, and undoubtedly the methodology and findings of this latest study will be controversial and debated. However, whatever the discussion, there are arguably more relevant questions that must be asked: Do we need another mortality survey in Iraq? What is its value to those left behind? Most importantly, what is the benefit of these surveys to policy-makers, and will they impact future decision-making?

## Winning Democracy, Gaining Lawlessness

Although Iraq was proclaimed a republic in 1958, military dictators ruled the country until 2003. The ousting of Saddam Hussein in the 2003 invasion and the subsequent occupation represent only a short chapter in the series of tragic events affecting Iraq. The profound public health impact of repeated conflict in the country's history has been critically examined by authors and academics alike. It is estimated that during 28 years of conflict (1980–2008), Iraq has lost approximately 2.5 million people [21].

However, living in Iraq today is no longer about how many have died, but how future deaths should be prevented. The results of the present and similar studies provide a picture of the living victims (war orphans, widows, and injured, disabled, and displaced people) whose health and social needs must be met. Sadly, for policy-makers in Iraq neither this new survey nor others will have much of an impact. Providing effective public services requires a strong government, but Iraq today, according to the Brookings Institution, is the world's fourth weakest state in political terms (after Somalia, Afghanistan, and the Democratic Republic of the Congo) [22].

The findings of Amy Hagopian and colleagues may help some families feel that their loss has at least been recognised, but continued sectarian bombings and targeted killings are deepening the sense of insecurity that continues to gnaw away at Iraq [23]. As a scientific community, yes, we want an accurate war tally that can be used as evidence against waging future wars, but people also want to live without fear of becoming a body counted by epidemiologists. In a region with escalating violence, sadly, this may be a distant dream.
